# Soy-Leaf Extract Exerts Atheroprotective Effects via Modulation of Krüppel-Like Factor 2 and Adhesion Molecules

**DOI:** 10.3390/ijms18020373

**Published:** 2017-02-10

**Authors:** Jong-Min Han, Hua Li, Moon-Hee Cho, Seung-Hwa Baek, Chul-Ho Lee, Ho-Yong Park, Tae-Sook Jeong

**Affiliations:** 1 Division of Life Science, Daejeon University, Daejeon 300-716, Korea; malius@dju.kr; 2 Industrial Bio-materials Research Center, Korea Research Institute of Bioscience and Biotechnology (KRIBB), Daejeon 305-806, Korea; leehua@kribb.re.kr (H.L.); chomh21@gmail.com (M.-H.C.); micro340@hanmail.net (S.-H.B.); hypark@kribb.re.kr (H.-Y.P.); 3 Laboratory Animal Resource Center, KRIBB, Daejeon 305-806, Korea; chullee@kribb.re.kr; 4 Department of Biomolecular Science, Korea University of Science and Technology, KRIBB, Daejeon 305-806, Korea

**Keywords:** adhesion molecule, atherosclerosis, inflammation, KLF2, soy leaf

## Abstract

Soy-leaf extracts exert their cardioprotective effects by inducing endothelium-dependent vasodilation in the arteries, and they favorably modulate the serum lipid profile. In this study, we investigated the atheroprotective effects of an ethanol extract of soy leaf (ESL) in human umbilical vein endothelial cells (HUVECs) and high-cholesterol diet (HCD)-fed low-density lipoprotein receptor deficient (LDLR^−/−^) mice. ESL induced the expression of Krüppel-like factor 2 (KLF2), an endothelial transcription factor, and endothelial nitric oxide synthase (eNOS), and suppressed the expression of vascular cell adhesion molecule-1 (VCAM-1) and intercellular adhesion molecule-1 (ICAM-1) through moderate inflammatory signal activation, not only in tumor necrosis factor-α (TNF-α)-stimulated HUVECs but also in 7-ketocholesterol (7-KC)-stimulated HUVECs. ESL supplementation reduced aortic lesion formation in Western diet-fed LDLR^−/−^ mice by 46% (*p* < 0.01) compared to the HCD group. ESL also markedly decreased the aortic expression levels of VCAM-1, ICAM-1, monocyte chemotactic protein-1 (MCP-1), TNF-α, IL-6, IL-1β, matrix metallopeptidase 9 (MMP-9), and fractalkine, while the expression of KLF2 was significantly increased. These results suggest that ESL supplementation has potential for preventing HCD-induced atherosclerosis effectively.

## 1. Introduction

Atherosclerosis is a chronic inflammatory disease involving the formation of arterial plaques, which are characterized by inflammatory infiltrates and lipid accumulation. One of the initial and key events in endothelium response to inflammatory stimuli is the expression of adhesion molecules such as vascular cell adhesion molecule-1 (VCAM-1), intercellular adhesion molecule-1 (ICAM-1), and E-selectin. These proteins mediate early leukocyte attachment and rolling on the endothelial surface [1].

Krüppel-like factor 2 (KLF2), an endothelial transcription factor, is a member of the zinc finger family of transcription factors. KLF2 suppresses the atherogenic/inflammatory signaling pathway in the endothelial cells and monocytes/macrophages [2,3]. A high KLF2 level markedly increases the expression of endothelial nitric oxide synthase (eNOS) and thrombomodulin (TM), while reducing cytokine-mediated activation of pro-inflammatory genes such as VCAM-1 in human endothelial cells [3,4,5]. Statins exert endothelial atheroprotective effects via KLF2 induction [6], and inhibit the expression of adhesion molecules, which in turn reduces inflammation by decreasing the number of immune-cells within the vessel wall [7,8]. This reduction in the expression of adhesion molecules and the consequential decrease in the immune-cell content of atherosclerotic lesions have been observed in animal models and human tissues [9]. KLF2 expression is induced by laminar shear stress, and its expression in the human aorta is robust in the linear segments of the arterial tree, but deficient at branch points, which are regions that are particularly prone to atherosclerotic lesion formation [10]. Recent studies using experimental animal models have shown that KLF2 plays an important role in maintaining atheroprotective and anti-inflammatory activities. Hemizygous state of KLF2 (KLF2^+/−^) increases diet-induced atherosclerosis in apolipoprotein E-deficient mice [11]. In contrast, the treatment of immune-deficient mice with KLF2-overexpressing monocytes significantly reduced carrageenan-induced acute paw edema in C.B-17-Scid-beige mice [5].

Oxidized low-density lipoproteins (LDLs) play a central role in atherosclerosis, and at least in part their toxicity is due to the formation of oxysterols, which have dual cytotoxic effects on the cells of the vascular wall through their ability to induce apoptosis in endothelial and smooth muscle cells, and necrosis in fibroblasts [12]. Oxysterols present in human atherosclerotic plaques play an active role in plaque development. The major oxysterols found in oxidized LDLs are 7α-hydroxycholesterol and 7-ketocholesterol (7-KC).

Soybean (*Glycine max* (L.) Merr.) is widely cultivated in Asia and in Western countries, and it is well known for its health protective properties against certain cancers, osteoporosis, atherosclerosis, and coronary heart disease [13,14]. The biologically active components in soybeans and soy products responsible for these beneficial effects are isoflavones and soy proteins [15,16]. Both soybeans and soy leaf is consumed in Asian countries. Soy leaf contains isoflavonols, their glycosides, isoflavones, pterocarpans, and pheophorbides, products of chlorophyll breakdown [17,18,19]. Soy-leaf extracts mainly containing kaempferol glycosides are cardioprotective because they induce endothelium-independent relaxation in rat carotid arteries [20], and they favorably modulate serum lipid profile [21]. We previously demonstrated that pterocarpan-enriched soy-leaf extract and soy-leaf extract containing kaempferol glycoside and pheophorbides improve glucose homeostasis in high-fat diet-induced type 2 diabetic mice and *db*/*db* mice, respectively [18,22]. Soy-leaf extracts increase plasma high-density lipoprotein-cholesterol (HDL-C) levels in overweight individuals [23], and improve blood glucose and dyslipidemia in pre-diabetic subjects [24]. Although dietary soy leaf is known to have beneficial effects on serum lipid profiles, carotid arteries, obesity, and diabetes, there are no reports on the anti-atherogenic effects of soy leaf via KLF2 induction in human umbilical vein endothelial cells (HUVECs) or atherosclerotic animal models. Thus, the purpose of this study is to investigate the effect of an ethanol extract of soy leaf (ESL) on the expression of adhesion molecules and inflammatory factors in HUVECs stimulated with tumor necrosis factor-α (TNF-α) or 7-KC. Moreover, this study provides evidence that KLF2 induction by ESL decreases the expression of VCAM-1 and ICAM-1 via moderate inflammatory signal activation in HUVECs, and reduces diet-induced atherosclerotic lesion formation in the aortic sinus of LDL receptor-deficient (LDLR^−/−^) mice.

## 2. Results and Discussion

### 2.1. ESL Inhibited the Adhesion of Monocytes to Activated HUVECs and Expression of Adhesion Molecules

**Figure 1:**
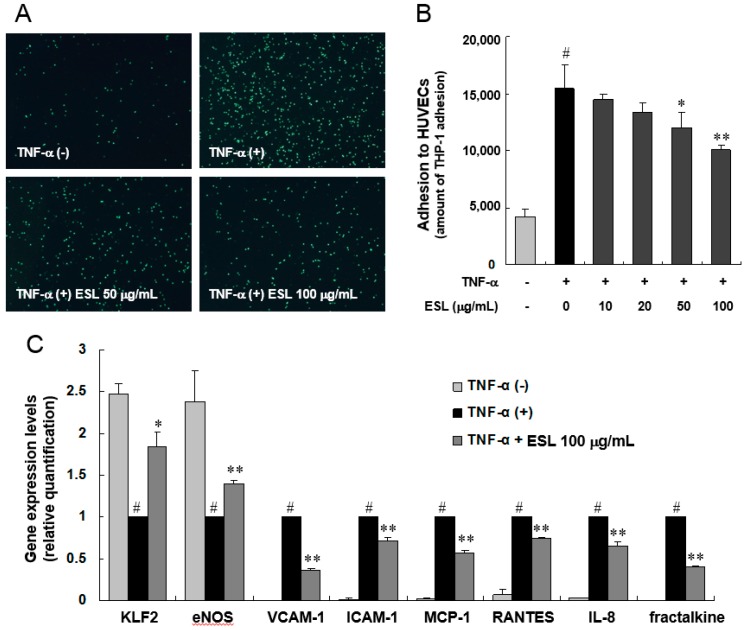
Effects of extract of soy leaf (ESL) on THP-1 monocyte adhesion and expression of atherogenic genes in TNF-α-stimulated human umbilical vein endothelial cells (HUVECs). (**A**) HUVECs were pretreated with or without ESL at the indicated concentrations for 2 h, and then stimulated with 10 ng/mL TNF-α for 8 h. Calcein AM-labeled THP-1 monocytes were added to each well for 1 h, the non-adherent cells were removed by rinsing, and adherent cells were visualized using fluorescence microscopy with a fluorescein blue filter (magnification, 40×); (**B**) The bar graph represents the amount of THP-1 adhesion to HUVECs; (**C**) TNF-α-induced expression of adhesion molecules and chemokines was quantified by real-time qRT-PCR as described in the Experimental Section. ^#^ *p* < 0.01 vs. the untreated group. * *p* < 0.05 and ** *p* < 0.01 vs. the TNF-α-treated group.

This study examined whether ESL regulates the adhesion of monocytes to TNF-α-stimulated HUVECs. The adhesion of THP-1 cells to HUVECs increased by approximately 4.4-fold following TNF-α stimulation (10 ng/mL), and the adhesion decreased in a dose-dependent manner following treatment with ESL (Figure 1A,B). Real-time quantitative reverse transcription-polymerase chain reaction (qRT-PCR) analysis of adhesion molecules, chemokines, and inflammation-associated genes was performed to investigate the molecular mechanism underlying the inhibitory effect of ESL on monocyte adhesion to TNF-α-stimulated HUVECs. The mRNA expression levels of VCAM-1, ICAM-1, monocyte chemotactic protein-1 (MCP-1), regulated upon activation normal T-cell expressed and secreted (RANTES), interleukin (IL)-8, and fractalkine in TNF-α-stimulated HUVECs were each reduced significantly (*p* < 0.01) when pretreated with 100 µg/mL of ESL. In addition, TNF-α-induced suppression of KLF2, a novel regulator of endothelial pro-inflammatory activation, and eNOS was reversed by pretreatment with ESL (Figure 1C). Taken together, we observed that ESL exert anti-inflammatory effect and activates KLF2 and eNOS. This result confirmed the correlation between KLF2 and inflammatory factors, suggesting that ESL is likely to regulate these correlations.

### 2.2. ESL Antagonized TNF-α-Induced Suppression of KLF2 and eNOS Expression

**Figure 2:**
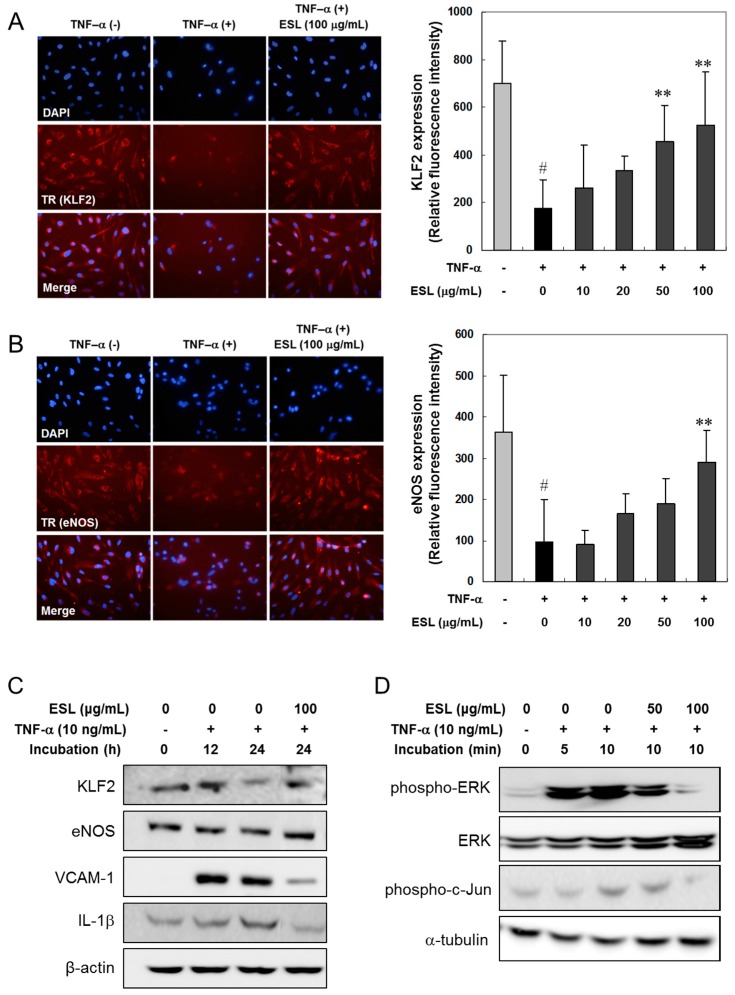
Effects of ESL on the suppression of KLF2 and eNOS, and Western blot analysis of target proteins and phosphorylation of ERK and c-Jun in TNF-α-stimulated HUVECs. (**A**,**B**) The cells were pretreated with or without ESL at the indicated concentrations for 2 h, and then stimulated with 10 ng/mL TNF-α for 24 h. The expression of KLF2 and eNOS was visualized by immunocytochemical (photographs, 400×) and immunofluorescence analyses. The cells were stained with antibodies directed against KLF2 and eNOS, and KLF2 and eNOS expression was detected with Texas Red (TR) or fluorescein isothiocyanate (FITC)-conjugated secondary antibodies using fluorescence microscopy. The bar graphs represent quantitative results obtained using a spectrofluorometer at excitation and emission wavelengths of 485 and 535 nm, respectively. ^#^ *p* < 0.01 vs. untreated group. ** *p* < 0.01 vs. the TNF-α-treated group; (**C**,**D**) The cells were pretreated with or without ESL at the indicated concentrations for 2 h and then stimulated with 10 ng/mL TNF-α for the indicated incubation times. The total cell extracts (**C**) and cytoplasmic extracts (**D**) were subjected to 10% SDS-PAGE and Western blot analysis with the respective primary antibodies.

The protein expression of KLF2 and eNOS was examined using immunofluorescence assays (Figure 2A,B), and confirmed using Western blot analysis (Figure 2C). TNF-α treatment of HUVECs strongly reduced the expression of endogenous KLF2 and eNOS (Figure 2A,B). ESL treatment significantly antagonized the suppressed expression of KLF2 and eNOS in a dose-dependent manner (Figure 2A,B). The protein levels of KLF2 and eNOS significantly decreased in HUVECs in response to treatment with 10 ng/mL TNF-α for 24 h (Figure 2C). However, ESL (100 μg/mL) treatment markedly reversed the TNF-α-induced suppression of KLF2 and eNOS protein expression. Additionally, the protein expression of VCAM-1 and IL-1β was markedly reduced in TNF-α-stimulated HUVECs following pretreatment of ESL (Figure 2C). To investigate the mechanism underlying the inhibitory effect of ESL on the expression of inflammatory mediators further, the effect of ESL on TNF-α-induced phosphorylation of ERK and c-Jun was examined by Western blot analysis. TNF-α caused a rapid and significant increase in the phosphorylation of ERK and c-Jun within 10 min, and pretreatment with 100 μg/mL ESL markedly suppressed this phosphorylation (Figure 2D).

### 2.3. ESL Antagonized 7-KC-Induced Suppression of KLF2 and eNOS Expression

**Figure 3:**
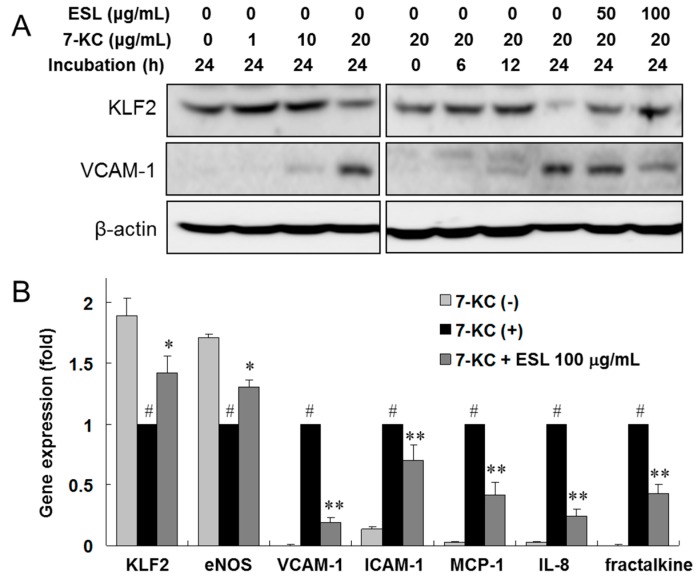

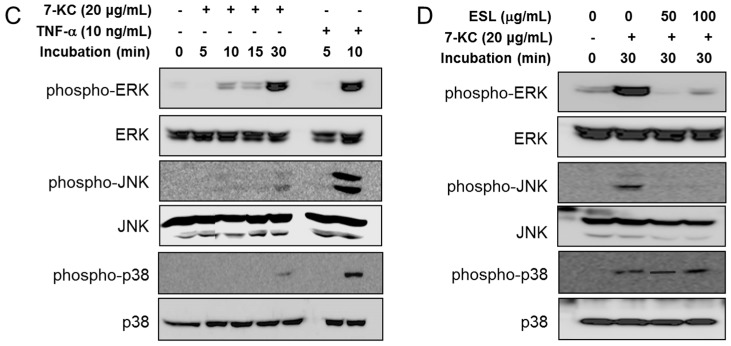
Effects of ESL on the expression of KLF2 and VCAM-1 proteins and atherogenic genes, and phosphorylation of mitogen-activated protein kinases (MAPKs) in 7-ketocholesterol (7-KC)-stimulated HUVECs. (**A**) The cells were pretreated with or without ESL for 2 h, and then stimulated with 7-KC in a dose- and time-dependent manner; (**B**) The mRNA levels of the indicated targets were determined by real-time qRT-PCR as described in the Materials and Methods Section. ^#^ *p* < 0.01 vs. untreated group. * *p* < 0.05 and ** *p* < 0.01 vs. the 7-KC-treated group; (**C**) The cells were stimulated with 7-KC or TNF-α for the indicated incubation times; (**D**) Cells were pretreated with or without ESL for 2 h, and then stimulated with 20 μg/mL 7-KC for 30 min. The protein levels were analyzed by Western blot with the respective primary antibodies and the corresponding phospho-specific antibodies. The total MAPK level was used as the internal control.

The results in Section 2.2 show that ESL inhibits adhesion molecule expression, adhesion of monocytes to endothelial cells, and suppression of KLF2 expression in TNF-α-stimulated HUVECs. Next, we investigated the effects of ESL on oxysterol-stimulated atherogenic gene expression in HUVECs. Confluent HUVECs were exposed to 7-KC, a major oxysterol, following pretreatment with or without ESL (0–100 μg/mL). After 24 h, KLF2 protein expression decreased in 7-KC-stimulated HUVECs compared to that in 7-KC-untreated cells (Figure 3A). However, ESL treatment antagonized the 7-KC-induced suppression of KLF2 protein expression in a dose-dependent manner. In contrast, VCAM-1 protein expression was markedly upregulated in 7-KC-stimulated HUVECs after 24 h, and ESL treatment dose-dependently inhibited 7-KC-induced VCAM-1 protein expression. To confirm the regulatory effect of ESL further, we examined the mRNA expression levels of adhesion molecules, chemokines, and inflammatory regulated genes using real-time qRT-PCR analysis. The increased mRNA expression of VCAM-1, ICAM-1, MCP-1, IL-8, and fractalkine induced by 7-KC was reduced significantly (*p* < 0.01), and the decreased expression of KLF2 and eNOS was reversed (*p* < 0.05) following pretreatment with 100 μg/mL of ESL (Figure 3B). Next, we examined the influence of ESL on mitogen-activated protein kinase (MAPK) signaling pathway to understand the underlying molecular mechanisms. The effects of ESL on 7-KC-stimulated phosphorylation of extracellular signal-regulated kinase (ERK) and c-Jun N-terminal kinase (JNK) in HUVECs were evaluated by Western blot analysis. 7-KC (20 μg/mL) significantly increased the phosphorylation of ERK and JNK, as well as p38 MAPK, within 30 min (Figure 3C). Pretreatment with ESL (50 and 100 μg/mL) markedly suppressed the activation of ERK and JNK, but the activity of p38 MAPK was not altered by ESL 2.1 sectiontreatment (Figure 3D).

### 2.4. ESL Reduced the Development of Atherosclerosis in LDLR^−/−^ Mice

**Figure 4:**
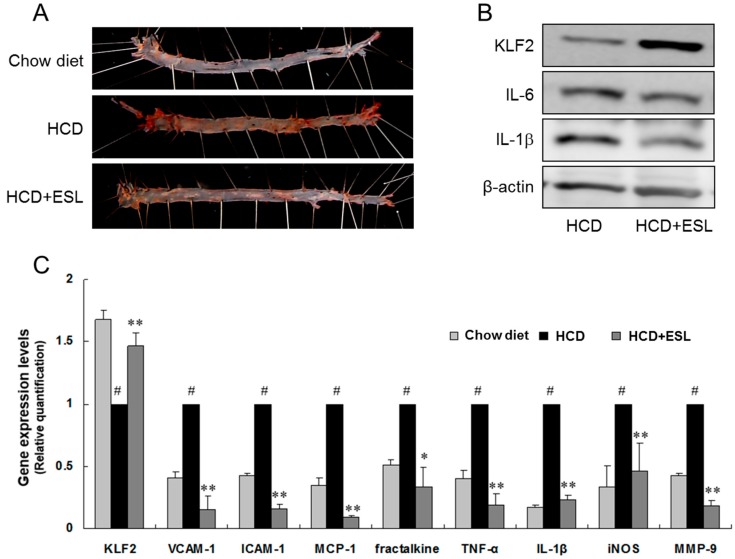
Effects of ESL on lipid deposition and expression of atherogenic markers in the aortas of LDLR^−/−^ mice fed a HCD for 12 weeks: (**A**) representative photographs of an oil red O-stained whole aorta (en face) from each group are shown; (**B**) the protein levels of KLF2, IL-6, and IL-1β were measured by Western blot analysis; and (**C**) the mRNA expression of the indicated targets was determined by real-time qRT-PCR. ^#^ *p* < 0.01 vs. the chow diet group. * *p* < 0.05 and ** *p* < 0.01 vs. the HCD group. Chow diet, chow-fed group; HCD, high-cholesterol diet-fed group; HCD + ESL, 1% (*wt*/*wt* diet) ESL supplemented HCD-fed group.

The effect of ESL on the formation of atherosclerotic lesions was evaluated in high-cholesterol diet (HCD)-fed LDLR^−/−^ mice by en face analysis. For the first 12 weeks, the atherosclerotic lesions were confined to the aorta in the HCD-fed LDLR^−/−^ mice (Figure 4). After 12 weeks, the atherogenic lesions extended to the thoracic and abdominal aortas. The atherogenic lesion area in the aortic arch and descending aorta increased markedly in the HCD group fed a HCD, compared to that in the chow diet group fed a normal chow diet. Comparison with the HCD group, the HCD + ESL group showed significantly decreased atherogenic lesion area (Figure 4A). Expression of KLF2 markedly increased, while that of IL-6 and IL-1β decreased in the HCD + ESL group compared to that in the HCD group (Figure 4B). The mRNA expression of adhesion molecules, chemokines, and cytokines (VCAM-1, ICAM-1, MCP-1, fractalkine, TNF-α, IL-1β, inducible nitric oxide synthase (iNOS), and matrix metallopeptidase 9 (MMP-9)) in the aortas from the HCD group increased markedly when compared with that in the chow diet group (Figure 4C). However, the expression of these genes significantly decreased (*p* < 0.01) in the HCD + ESL group. In particular, the suppressed mRNA and protein expression of KLF2 in the HCD group was restored in the HCD + ESL group (Figure 4B,C).

**Figure 5:**
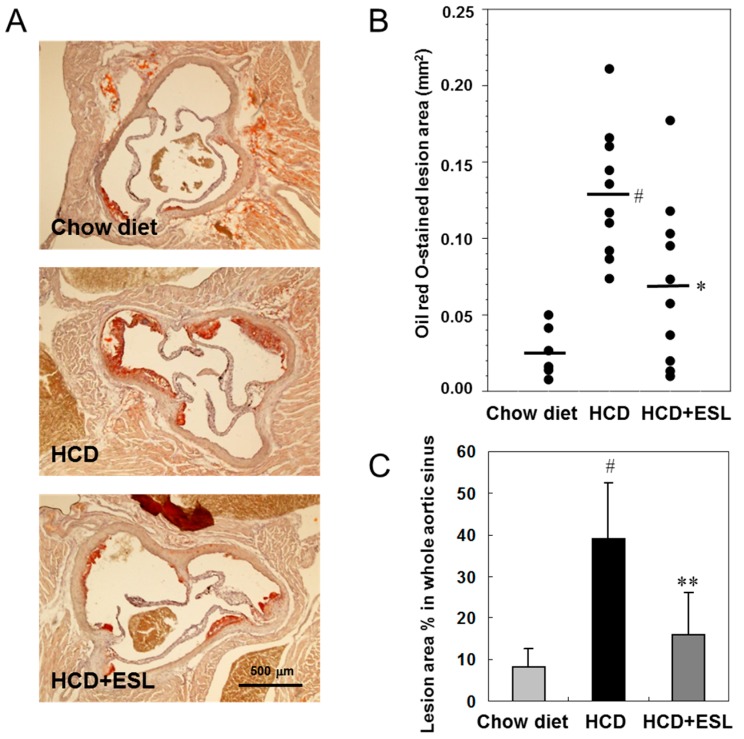
Effects of HCD + ESL on atherosclerotic lesion formation in the aortic sinus of LDLR^−/−^ mice fed a Western diet for 12 weeks: (**A**) representative micrographs of an oil red O-stained cross-section of an aortic sinus (magnification, 40×) from each group are shown; (**B**) Quantification of the oil red O-stained aortic sinus lesional areas. Black lines are shown as mean lesion area; and (**C**) the percentage of oil red O-stained lesion areas in the whole aortic sinus from each group was performed by computer-associated morphometry and the results are presented in the graph. The results are shown as mean ± SD. ^#^ *p* < 0.01 vs. the chow diet group. * *p* < 0.05 and ** *p* < 0.01 vs. the HCD group. Chow diet, chow-fed group; HCD, high-cholesterol diet-fed group; HCD + ESL, 1% (*wt*/*wt* diet) ESL supplemented HCD-fed group.

Next, the atherosclerotic lesions in the Western diet-fed or chow-fed LDLR^−/−^ mice were identified using oil red O to stain frozen cross-sections of the aortic sinus (Figure 5A). The mean oil-red O-stained lesion area was (70.3 ± 53.9) × 10^3^ μm^2^ in the HCD + ESL group (*p* < 0.05) and (129.6 ± 45.7) × 10^3^ μm^2^ in the HCD group (Figure 5B). The atherogenic lesion area in the whole aortic sinus was decreased significantly by 59% (*p* < 0.01) in the HCD + ESL group compared with that in the HCD group (Figure 5C).

It is well known that soy proteins reduce the serum total cholesterol levels and risk of atherosclerosis [25]. In addition, specific soy components including isoflavones, amino acid constituents, saponins, fiber, and trypsin inhibitors may contribute to the cholesterol-lowering effect of soy protein, either directly or indirectly [26].

**Figure 6:**
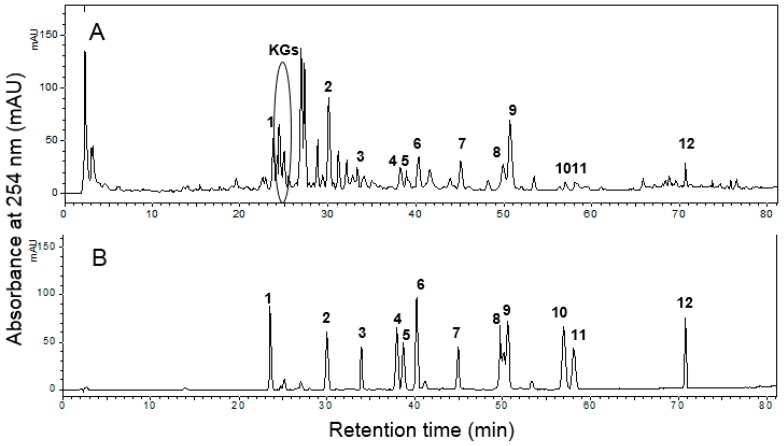
HPLC profiles of: ESL (**A**); and the internal standard mixture (**B**). Description of the peaks: **1**, daidzin; **2**, genistin; **3**, apigenin-7-*O*-β-glucoside; **4**, ononin; **5**, genistin-5,4′-methyl ether; **6**, daidzein; **7**, glyceofuran; **8**, isotrifoliol; **9**, coumestrol; **10**, formononetin; **11**, afromosin; **12**, phaseol; KGs, kaempferol glycosides. In the internal standard mixture (**B**), the concentrations of **1**, **3**, **4**, **5**, **6**, **9**, **10**, and **11** were 30 μg/mL; the concentrations of **2** and **12** were 20 μg/mL; the concentration of **7** was 130 μg/mL; and the concentration of **8** was 70 μg/mL.

ESL and standard compounds were analyzed using HPLC diode-array detector (DAD) at 254 nm (Figure 6). Unlike the components of soybean, which are mainly daidzein, genistein, daidzin, genistin, and their derivatives, ESL primarily contained kaempferol glycosides (KGs, retention time (*R*t) = 24 to 35 min), isoflavones (daidzin and genistin), and pterocarpans (glyceofuran, isotrifoliol, coumestrol, and phaseol) (Figure 6A). Pterocarpans were either absent or present only in very small amounts in soybean [19]. Although there have been many studies on the beneficial effects of soybean and dietary soy products on the cardiovascular system, few reports about the vascular activity of soy leaf are present [20,21].

In this study, ESL significantly suppressed the early stage of atherosclerotic development in the aortas of LDLR^−/−^ mice fed a CHD. The reduction of atherosclerotic progression and macrophage accumulation was accompanied by the suppressed expression of adhesion molecules in LDLR^−/−^ mice and in HUVECs. Moreover, we demonstrated that the anti-atherogenic mechanism of ESL leads to the inhibition of VCAM-1, ICAM-1, and chemokine expression as well as KLF2 upregulation in TNF-α-stimulated or 7-KC-stimulated HUVECs, and the aortas of HCD-fed LDLR^−/−^ mice.

TNF-α and IL-1β have been shown to induce the activation of endothelial cells, resulting in the coordinated up-regulation of VCAM-1, ICAM-1, and E-selectin expression [27]. Adhesion of inflammatory cells to the vascular endothelium is followed by transendothelial migration of these cells to the arterial intima. Chemokines including MCP-1, RANTES (CCL5), fractalkine (CX3CL1), MIP1-α (CCL3) and MIP1-β (CCL4), which drive leukocyte transendothelial migration, have been implicated in the initiation and progression of atherosclerosis. In the present study, we clearly demonstrated that ESL inhibits THP-1 cell adhesion to TNF-α-stimulated HUVECs. The mRNA expression of adhesion molecules and chemokines tested in this report, including VCAM-1, ICAM-1, MCP-1, RANTES, IL-8, and fractalkine, was also down-regulated by ESL treatment. Therefore, the inhibitory effect of ESL on the expression of molecules presented in this report demonstrates a new mechanism responsible for the atheroprotective activity of ESL.

KLF2 is a potent regulator of eNOS, and contributes to the pro-inflammatory activation of endothelium in animal models and human cell lines. In human cell studies using HUVECs, treatment with cytokines such as TNF-α and IL-1β reduces KLF2 expression, and forced overexpression of KLF2 prevents cytokine-mediated induction of adhesion molecules such as VCAM-1 and E-selectin [3]. Notably, a relative lack of KLF2 expression was detected in the endothelial cell lining of bifurcations, a region prone to developing atherosclerotic lesions in the human aorta [10]. In our experiments using HUVECs, the treatment of these cells with TNF-α or 7-KC reduced the expression of KLF2 and eNOS, whereas, the expression levels of VCAM-1, MCP-1, and IL-8 were elevated. However, ESL restored the expression of KLF2 and eNOS. In addition, it was able to inhibit the atherogenic mechanism. Some statins, including simvastatin, cerivastatin, and lovastatin, have been shown to induce endothelial KLF2 expression in patients, at pharmacological concentrations [6]. These statins reduce the proliferative and functional responses of T cells and endothelial cells, and induce higher levels of KLF2 expression in these two cell types [28,29]. These results are important for understanding the therapeutic mechanisms of KLF2 as a potentially important target for the treatment of diseases in which pro-inflammatory T cells play a crucial role, including atherosclerosis, myocarditis, and various autoimmune diseases [7]. In this study, we demonstrated that Western diet-induced atherosclerotic development in LDLR^−/−^ mice was accompanied by decreased levels of KLF2 and increased levels of atherogenic mediators such as VCAM-1, ICAM-1, MCP-1, TNF-α, and IL-1β by using experimental animal models. However, ESL treatment decreased the expression of these genes; in particular, the mRNA and protein expression of KLF2 recovered significantly in ESL-treated mice. Therefore, pharmacological manipulation of KLF2 expression may be an effective therapeutic strategy for the treatment of atherosclerotic development in human cells and experimental animal models.

Oxysterols, especially 7-KC or 7β-hydroxycholesterol, are present at relatively high concentrations in refined oxidized LDL in vitro and in high abundance in arterial foam cells and atherosclerotic plaques in vivo [12]. 7-KC, a major oxysterol, has potent pro-inflammatory and cytotoxic activities, and it can induce monocyte-endothelial cell interaction via up-regulation of VCAM-1/ICAM-1 on the endothelial cells [30]. This study also demonstrated that ESL markedly inhibited 7-KC-induced expression of atherogenic genes, including VACM-1, MCP-1, IL-8, and fractalkine in HUVECs, and the suppressed KLF2 and eNOS expression was restored following ESL treatment in HUVECs. In previous studies, we reported that pterocarpan-derived species have potent LDL-antioxidant activities and human acyl-coenzyme A: cholesterol acyltransferase 1 (hACAT-1) and hACAT-2 inhibitory activities [31,32]. These activities may be related to the atheroprotective effects of ESL in TNF-α- or 7-KC-stimulated HUVECs and LDLR^−/−^ mice. The results of this study also suggest that the beneficial effects of kaempferol glycosides in ESL may be owing to reduced endothelial damage in cardiovascular disease [33]. In recent studies, ESL supplementation with kaempferol glycosides and pterocarpans in overweight and obese individuals increased the plasma HDL-C level and ratio of HDL-C to TC, which is associated with a lower risk of atherosclerosis [23,24].

## 3. Materials and Methods

### 3.1. Preparation of ESL and HPLC Analysis

Soybeans, *G. max* (L.) Merr., were cultured in Jinju City (Gyeongsangnam-do, Korea) for 16 weeks. Air-dried leaves (500 g) were chopped and extracted twice with 95% ethanol (5 L) at room temperature for 48 h. The combined ethanol extract (ESL) was filtered through a 0.45-μm poly(tetrafluoroethylene) (PTFE) filter (Whatman International Ltd., Maidstone, UK) and concentrated and lyophilized in vacuo to yield a dark brown powder (90.5 g). The lyophilized ESL powder was then dissolved in methanol and filtered through a 0.45-μm PTFE filter for HPLC analysis.

The main polyphenol compounds present in the ESL were analyzed and confirmed using an analytical HPLC system (Shimadzu HPLC system equipped with a binary pump delivery system, a DAD, and an autosampler, Shimadzu Corp., Tokyo, Japan; Cosmosil 5C18-MS-II column, 4.6 mm × 150 mm, 5 μm, Nacalai Tesque, Japan; injection volume: 10 μL; mobile phase: 0.1% acetic acid in water (solvent A) and acetonitrile (solvent B); a linear gradient elution program was used: 5%–40% B for 0–60 min, 40%–100% B for 60–80 min, 100%–5% B for 80–100 min; flow rate: 1 mL/min; absorbance: 254 nm). The external standards for apigenin-7-*O*-β-glucoside, coumestrol, daidzein, daidzin, formononetin, genistin, and ononin were obtained from Sigma-Aldrich, while those for afromosin, genistin-5,4′-methyl ether, glyceofuran, isotrifoliol, phaseol, and kaempferol glycosides (KGs) were isolated from ESL [18,19]. HPLC profiles of ESL and the standard mixture are shown in Figure 6A,B.

### 3.2. Materials and Cell Culture

7-Ketocholesterol (7-KC), 2′,7′-*bis*-(2-carboxyethyl)-5-(and-6)-carboxyfluorescein acetoxymethyl ester (BCECF-AM), and 4′-6-diamidino-2-phenylindole (DAPI) were obtained from Sigma-Aldrich (St. Louis, MO, USA). Recombinant human TNF-α was purchased from Strathmann Biotechnology (Hannover, Germany). BulletKit endothelial cell basal medium-2 (EBM-2) was purchased from Lonza Bioscience (Verviers, Belgium). Anti-KLF2, anti-eNOS, anti-VCAM-1, and anti-actin polyclonal antibodies were obtained from Santa Cruz Biotechnology (Santa Cruz, CA, USA). Texas Red (TR) or fluorescein isothiocyanate (FITC)-conjugated secondary antibodies were obtained from Abcam (Cambridge, MA, USA). 2′,7′-dichlorofluorescein diacetate was purchased from Molecular Probes (Eugene, OR, USA).

HUVECs (Lonza Bioscience) were grown in EBM-2 on gelatin-coated culture dishes or six-well plates, and used for the experiments within the first three to five passages. The cells were grown in monolayers at 37 °C in a humidified atmosphere of 5% CO_2_ and 95% air, and were used for experiments at >80% confluence. THP-1 human acute monocytic leukemia cells obtained from the American-Type Culture Collection (Rockville, MD, USA) were cultured in RPMI-1640 medium (Invitrogen, Carlsbad, CA, USA) supplemented with 10% (*v*/*v*) fetal bovine serum (FBS, Lonza Bioscience) and penicillin–streptomycin mixture (Lonza Bioscience), under the same conditions used for HUVECs.

7-KC is prepared with 20 mg/mL stock in ethanol according to the manufacturer’s method, and then diluted with PBS to the desired concentration. ESL is dissolved in dimethyl sulfoxide (DMSO) were added to the medium. The final concentration of DMSO did not exceed 0.05% (*v*/*v*). To exclude the contamination of endotoxin in ESL, the endotoxin levels in ESL were detected using a Limulus Amoebocyte Lysate assay (Catalog No. 50-647U, Lonza Walkersville, Inc., Walkersville, MD, USA). These levels were very low (<0.1 EU endotoxin/mL).

### 3.3. Animal Experiments

Homozygous LDL receptor deficient (LDLR^−/−^, C57BL/6J background) mice were purchased from The Jackson Laboratory (Bar Harbor, ME, USA) and housed at the Korea Research Institute of Bioscience and Biotechnology (KRIBB, Daejeon, Korea). Ten-week-old male LDLR^−/−^ mice (*n* = 26) were randomly divided into three groups. The HCD group (*n* = 10) was fed high-cholesterol diet (No. 100244; Dyets Inc., Bethlehem, PA, USA) composing 0.15% cholesterol by weight, with no cholic acid. The composition of this diet in g/kg was: casein, 195; d,l-methionine, 3; cornstarch, 150; sucrose, 341.46; cellulose, 50; milk fat, 210; salt mix, 35; vitamin mix, 10; calcium carbonate, 4; cholesterol, 1.5; and ethoxyquin, 0.04. The HCD + ESL group (*n* = 10) was fed a HCD supplemented with 1% (*wt*/*wt* diet) ESL for 12 weeks. Additionally, this study used chow-fed LDLR^−/−^ mice (*n* = 6, Chow diet group) as a negative control. The animal study protocols were approved by the Institutional Animal Care and Use Committee of KRIBB (KRIBB-AEC-080215).

### 3.4. Measurement of Fatty Streak Lesion Area

For en face analysis, the aortas were opened longitudinally and pinned flat onto black rubber plates. Each aorta was fixed with 10% neutral-buffered formalin for 24 h. After fixation, the aorta was washed once with PBS, and then stained with oil red O solution for 30 min. Excess stain removed by rinsing with 60% propylene glycol (Sigma-Aldrich). Images of oil red O-stained aortas were taken with a Sony digital camera (SONY Corporation, Tokyo, Japan).

Aortic roots were embedded in Tissue-Tek OCT compound (Sakura Finetek, Inc., Torrance, CA, USA), and placed on a cryotome (model AS620; Shandon, Pittsburgh, PA, USA). Cryostat sections of the aortic sinus (10 μm) were collected and stained with oil red O. The aortic images were captured with a BX61 microscope (Olympus, Tokyo, Japan), and atherosclerotic lesions were quantified by computer image analysis using Metamorph imaging software (Molecular Devices, Sunnyvale, CA, USA).

### 3.5. Cell Adhesion Assay

HUVECs growing in 96-well culture plates were pretreated with or without ESL for 2 h, and then stimulated with 10 ng/mL TNF-α for 8 h. THP-1 cells were labeled with BCECF-AM fluorescent dye at a concentration of 10 μM, at 37 °C for 1 h. The labeled cells were harvested by centrifugation, washed three times with PBS before suspension in the medium, and added to the HUVECs. Co-incubation was done at 37 °C for 1 h, and unbound THP-1 cells were removed by stringently washing them four times with medium (RPMI-1640, 1% FBS) and twice with phosphate buffered saline (PBS). An image of THP-1 cell distribution was obtained with an ECLIPSE 2000 fluorescent microscope (Nikon, Tokyo, Japan). THP-1 cells bound to HUVECs were lysed in lysis buffer containing 50 mM Tris-HCl (pH 8.0) and 0.1% (*w*/*v*) sodium dodecyl sulphate (SDS). The fluorescence intensity was measured on a Wallac 1420 spectrofluorometer (Perkin-Elmer, Turku, Finland; Excitation wavelength = 488 nm, Emission wavelength = 535 nm).

### 3.6. Immunofluorescence Analysis

The expression of KLF2 and eNOS in HUVECs was detected using a cell enzyme-linked immunosorbent assay. Briefly, HUVECs growing in 96-well culture plates were treated with or without ESL for 2 h, and then stimulated with 10 ng/mL TNF-α for 24 h. The cells were washed with PBS, and fixed with 4% (*v*/*v*) paraformaldehyde at 4 °C for 30 min. They were then incubated with anti-KLF2 or anti-eNOS antibodies, and stained with the appropriate secondary antibody conjugated to TR or FITC. Images were captured by fluorescence microscopy using a TR-conjugated secondary antibody. DAPI stain was used to visualize the nuclei of HUVECs. Fluorescence photographs were obtained with a BX-61 fluorescence microscope (Olympus). The expression of KLF2 and eNOS was measured with a Wallac 1420 spectrofluorometer using an FITC-conjugated secondary antibody.

### 3.7. Real-Time qRT-PCR Analysis

Total RNA was extracted from HUVECs and mouse aortas using an RNeasy RNA extraction kit (Ambion, Austin, TX, USA), and cDNA was synthesized with Omniscript RT kit (Qiagen, Santa Clarita, CA, USA) using oligo (dT) primers, according to the manufacturer’s instructions. PCR amplifications were quantified using the SYBR Green PCR Master Mix on a 7500-Real-Time PCR system (Applied Biosystems, Foster City, CA, USA). The primer sequences are summarized in Table 1.

All of the primer sets produced amplicons of the expected size, and their identities were verified by sequencing. The cycling conditions were 95 °C for 10 min, followed by 40 cycles of 95 °C for 10 s, 60 °C for 20 s, and 72 °C for 33 s. To detect and eliminate the possible primer-dimer artifacts, a dissociation curve was generated by adding a cycle of 95 °C for 15 s, 60 °C for 1 min, and 95 °C for 15 s. The results were normalized using actin or GAPDH as a reference gene, and represented as fold changes versus the reference gene.

### 3.8. Western Blot Analysis

Whole proteins from mouse aortas and harvested cells were extracted using radioimmunoprecipitation assay (RIPA) buffer. Cytoplasmic extracts of HUVECs were extracted using a hypotonic buffer (1 M HEPES, pH 7.9, 1 M MgCl_2_, 2.5 M KCl, 1 M DTT, and protease inhibitors). Mouse aortic tissue extracts (IL-6, IL-1β, and KLF2), total cell extracts (eNOS, KLF2, IL-1β, and VCAM-1), and cytoplasmic extracts (ERK, phospho-ERK, JNK, phospho-JNK, P38, and phospho-p38) were fractionated by electrophoresis on 10% SDS-PAGE gels, and transferred onto nitrocellulose membranes. The membranes were blotted with antibodies specific to each protein. The peroxidases bound to the blots were detected with the Immobilon Western HRP detection reagent (Millipore, Billerica, MA) using an Image Reader (LAS-3000 Imaging System; Fuji Photo Film, Tokyo, Japan).

### 3.9. Data Analysis

Data are expressed as mean ± SD values. Statistical significance (*p* < 0.05) between two groups was determined from two parallel experiments using the Student’s *t*-test.

## 4. Conclusions

The present study shows for the first time that ESL regulates the expression of KLF2 and inflammatory factors, and inhibits atherosclerotic processes in the endothelial cells and LDLR^−/−^ mice. These effects are apparently mediated by the down-regulation of proatherogenic genes, including adhesion molecules, chemokines, and pro-inflammatory mediators. These findings suggest that ESL may be useful as a dietary intervention for atherosclerosis.

## Figures and Tables

**Table 1:** Sequences of primers used in real-time qRT-PCR analysis.

Gene (Number)	Primer Sequence (Forward 5′–3′)	Primer Sequence (Reverse 5′–3’)
*hCCL5* (NM_002985)	CGGGAGTACATCAACTCTTTGGA	CAAGCTAGGACAAGAGCAAGCA
*heNOS* (NM_000603)	CTCATGGGCACGGTGATG	ACCACGTCATACTCATCCATACAC
*hFractalkine* (NM_002996)	CGCAGCATATTCAGGAAGCT	TCCTTGTCCATGTCCTGCTT
*hICAM-1* (NM_000201)	AGAGGTTGAACCCCACAGTC	TCTGGCTTCGTCAGAATCAC
*hIL-8* (NM_000584)	CCTAGATATTGCACGGGAGA	GTGGAACAAGGACTTGTGGA
*hKLF2* (NM_016270)	CCTCCTTGACGAGTTTTGTTTTTC	AAGGCATCACAAGCCTCGAT
*hMCP-1* (NM_002982)	GCTCAGCCAGATGCAATCAA	CTTGGCCACAATGGTCTTGA
*hVCAM-1* (NM_001078)	GTTGAAGGATGCGGGAGTAT	TTCATGTTGGCTTTTCTTGC
*hActin* (NM_001101)	GGCACCACACCTTCTACAAT	TCTGGGCATCCTTCACAGCT
*mFractalkine* (NM_009142)	TCACGTGCAGCAAGATGACA	TCCTTGACCCATTGCTCCTT
*mICAM-1* (NM_010493)	GTGATGCTCAGGTATCCATC	GAATACACGGTGATGGTAGC
*mIL-1β* (NM_008361)	ATGAGGACATGAGCACCTTC	CATTGAGGTGGAGAGCTTTC
*miNOS* (NM_010927)	GGCAGCCTGTGAGACCTTTG	TGCATTGGAAGTGAAGCGTTT
*mKLF2* (NM_008452)	CGCCACACATACTTGCAGCTA	GTGTACGCAGATGCGCCTTT
*mMCP-1* (NM_011333)	TGCTGACCCCAAGAAGGAAT	TGCTTGAGGTGGTTGTGGAA
*mMMP-9* (NM_013599)	CAGCCAACTATGACCAGGAT	TCGCTGGTACAGGAAGAGTA
*mTNF-α* (NM_013693)	CTCAGATCATCTTCTCAAAATTCGAGTGACA	CTTCACAGAGCAATGACTCCAAAGT
*mVCAM-1* (NM_011693)	CTGTTTGCAGTCTCTCAAGC	CCAAACACTTGACTGTGACC
*mGAPDH* (NM_008084)	ACATCATCCCTGCATCCACT	AGATCCACGACGGACACATT
